# Some Interesting Cases from the Medical Service at the Cattaraugus County Almshouse

**Published:** 1894-01

**Authors:** Clarence King

**Affiliations:** Machias, N. Y., Attending Physician Cattaraugus County Almshouse


### SOME INTERESTING CASES FROM THE MEDICAL SERVICE AT THE CATTARAUGUS COUNTY ALMSHOUSE.

By CLARENCE KING, M. D., Machias, N. Y.,

Attending Physician Cattaraugus County Almshouse.

Having been the attending physician at the Cattaraugus County Almshouse for a period of eight years, and having met with many interesting cases, I have thought some of them might possibly be of interest to others. As a preface to my paper, I would say that the institution now contains only about sixty or seventy inmates, less than one-half its population before the insane were removed to the State Hospital a year ago. But those that remain are all invalids, or of weak physical or mental strength, by reason either of disease or accident, of old age or of idiocy, or other mental disease. The hospital always contains patients under treatment, but, of course, at some seasons of the year there is much more sickness than at other times. Since my term of service began, we have had but one epidemic, excluding the cases of colds and coughs which are present in great number each Winter. This occurred in January, 1892, when nearly every inmate and attendant was attacked with

### INFLUENZA, OR LA GRIPPE.

The disease began in a ward on the third floor, occupied mostly by idiotic and half-witted men, and was evidently brought to the institution by a convalescing patient who came in a few days before and was placed in this room. The disease was confined to this ward for about ten days, every person being more or less sick with it, after which it appeared in other wards, but was not very severe, and only one death occurred. That was an old man with valvular disease of the heart who had pneumonia consecutive to grippe.

Influenza has long been regarded as an infectious disease, the poison being diffused through the air, and capable of being carried long distances. Although there is abundant proof in support of this view, yet I would cite this case as apparently antagonistic to the generally accepted opinion. The two preceding Winters, influenza of a severe form raged in Machias and the surrounding country, but not a single case occurred at the Almshouse, which is, to a certain extent, isolated during the Winter months. During these two Winters no case of influenza was brought in, except those far advanced in convalescence, or apparently well.

The third epidemic which visited Machias was mild in degree, yet, when introduced into the Almshouse, it readily followed the channels of communication between the different wards and buildings in the same way other diseases would have done which require actual contact, or close association for some time, in order to propagate them.

### HEREDITARY CHOREA.

At least four patients with hereditary chorea have been inmates at this institution. This disease was first described by Dr. Waters in 1841, but did not receive much attention until 1872, when Dr. George Huntington referred to it briefly in a general paper on Chorea. In 1885, my thesis on this disease, presented to the University of Buffalo, was published in the *New York Medical Journal*, and since that time quite an extensive literature has grown up. These cases of hereditary chorea occurred in two different families, both of which I have reported in full in other places,[1] and to which I would refer those interested in the disease. One of these patients is still here, slowly growing worse and more helpless year by year. But one case of Suydenham's chorea has been under treatment here within the last eight years, and that promptly recovered.

### SUB-OCCIPITAL ABSCESS.

Male, Irish, aged seventy-seven. Patient stated that he was run over by a wagon, the wheel passing over the back of his neck, immediately below the occipital protuberance. Pain and tender-

1. *Medical Press of Western New York*, December, 1886, and *Philadelphia Medical News*, July 13, 1889.

ness followed. Abscess developed slowly, which I incised some two weeks later. Had pain in top of head, and persisted in lying flat on his face, thus preventing drainage, and would allow no dressings on neck for a period of nearly two weeks. I made many punctures to a depth of an inch or more, and later made four or five incisions, each about an inch and a half long and an inch deep. Patient died of exhaustion about six weeks after date of injury. I think the proper treatment in this case would have been to have anesthetized him early, turned up large flaps, and scraped out all necrosed tissue, but he would not consent to it.

In February, 1891, seven patients in one ward, all old women and feeble, were taken sick with

### ACUTE INTESTINAL CATARRH.

Other cases of diarrhea followed in this same ward, and five patients died, three of them on the same day. Diarrhea did not occur in other wards, although the same food was given other patients, and the same conditions existed, apparently, in at least three other wards. The plumbing, however, was bad in these wards, particularly so in the closet communicating with the infected ward, where the floor was habitually wet from a leaky water-pipe. No case of typhoid fever has originated in the institution since I have been connected with it, but several cases have been brought here during convalescence, one of which suffered a relapse.

### FIBROMA MOLLUSCUM.

Male, aged seventy-seven; five feet two inches in height; weight, 135 pounds. Had always been feeble in health. At about thirty years of age noticed numerous soft tumors growing on his hands and face, which grew to the size of a pea or bean. At the time he came under my care he was literally covered from head to foot with these tumors, none of them larger than an English walnut. It would have been impossible to count them, but there must have been several thousand of them. This case I have reported more at length in the *American Medico-Surgical Bulletin* for July, 1893.

Syphilis is always present in some of its forms, and cancer is quite common. One case of cancer of the antrum, and another of the lip following an operation for the removal of an epithelioma, were unpleasant cases which we had to care for during several weeks. Another case, a

CANCER OF THE KIDNEY,

I wish to mention on account of the *post-mortem* findings. The patient was a German, aged eighty years, who had a well-marked blowing heart murmur, but was quite active for an old man. Three months before his death he began to have pain in his bowels and back; he emaciated rapidly, and developed a cancerous cachexia. He could not eat, had diarrhea alternating with constipation, and finally passed pinkish or blood-stained urine involuntarily. At the autopsy we found the right kidney cancerous, with a growth the size of a man's fist between the kidney and the bodies of the vertebræ, adherent to the vertebræ; also a calculus the size of a marble projecting from the upper surface of the liver at least one-half its size. This stone was easily removed by cutting the peritoneal covering and making compression to its base. It was hard, smooth, oblong in shape, and white in color. I believe it is rare to find such concretions in the liver, and still more so to find them projecting from its surface.

PARTIAL DISLOCATION OF CERVICAL VERTEBRÆ.

Male, aged sixty, lame, and walked with a cane. While descending stairs, fell and struck on his head, forcibly flexing the neck and turning chin toward right shoulder. When picked up, was unable to raise his head. Had sharp pain in back of neck, which persisted for some time, and was aggravated by every effort to raise the chin. I could not rotate the head in the least, and the fifth cervical vertebra was very prominent. I could not reduce the dislocation by moderate pressure applied direct to the displacement, and he refused more radical measures after the dangers were explained to him. He finally recovered, with inability to move the head from its flexed and rotated position.

SUDDEN DEATH FROM UNKNOWN CAUSE.

Male, Irish, age about thirty-five. Patient suffered an amputation of right leg at junction of middle and lower thirds. Operation done a few days before he came here. Pus formed in the wound, and I had to remove a few stitches to secure drainage. Subsequently, all went well and patient recovered sufficient to sit up and be dressed, a small surface of granulations, however, not being as yet covered by skin. One day, while sitting at a table playing cards, he spoke of a sudden bad feeling, and immediately fell to the floor and died without saying another word. His companions stated that he did not live three minutes after he made complaint. No autopsy was held.

### MOTOR APHASIA FOLLOWING APOPLEXY.

Male, Italian, age about fifty ; an inmate here about eight years. Previous to his coming here had an apoplectic stroke with paralysis of right arm and leg, and loss of voice. Has recovered use of leg so he is able to walk, but drags the right foot. Has no use of right arm, but carries it flexed at a right angle across his stomach, and straightens it only with difficulty, and with the aid of the other hand. He never has recovered his voice, with the exception of one word, "Yes," which he says in reply to any and every question. He understands everything, apparently, that is said to him, and will obey simple orders promptly. Is able to write with difficulty with left hand. With his other troubles he has had a chronic urethral discharge. About three years ago he had inflammation of the scrotum, which became swollen to twice the normal size and very painful. I made several punctures, from which oozed bloody serum, and afterward made incisions through skin to relieve the tension. But the whole anterior covering of the testicles sloughed away, leaving those organs completely exposed. However, granulations quickly formed after the slough separated, and the scrotal wall was rapidly repaired, with the exception of a fistula, which remained, and from which still comes a scanty purulent discharge.

This patient has a urethral stricture, which was probably the cause of this last mentioned affliction.

---

Some Experiments with Terraline.—Dr. C. H. Stowell, of Washington, D. C., concludes, after a series of experiments on the lower animals, that terraline is emulsified and absorbed the same as any other oil. It is a different product of petroleum than vaseline. The whole article describing these experiments, and the conclusion derived from them, can be found in the November number of *Food*, of which Dr. Stowell has recently assumed the editorship.

